# Out-of-Distribution Detection and Radiological Data Monitoring Using Statistical Process Control

**DOI:** 10.1007/s10278-024-01212-9

**Published:** 2024-09-16

**Authors:** Ghada Zamzmi, Kesavan Venkatesh, Brandon Nelson, Smriti Prathapan, Paul Yi, Berkman Sahiner, Jana G. Delfino

**Affiliations:** 1 Office of Science and Engineering Laboratories, Center for Devices and Radiological Health, U.S. Food and Drug Administration, 10903 New Hampshire Ave, Silver Spring, MD 20993 USA; 2 Department of Biomedical Engineering, Johns Hopkins University, 400 N. Charles St., Baltimore, MD 21218 USA; 3 University of Maryland School of Medicine, 685 W. Baltimore St., Baltimore, MD 21201 USA

**Keywords:** Out of distribution detection, Data drift monitoring, Statistical process control, Medical imaging

## Abstract

Machine learning (ML) models often fail with data that deviates from their training distribution. This is a significant concern for ML-enabled devices as data drift may lead to unexpected performance. This work introduces a new framework for out of distribution (OOD) detection and data drift monitoring that combines ML and geometric methods with statistical process control (SPC). We investigated different design choices, including methods for extracting feature representations and drift quantification for OOD detection in individual images and as an approach for input data monitoring. We evaluated the framework for both identifying OOD images and demonstrating the ability to detect shifts in data streams over time. We demonstrated a proof-of-concept via the following tasks: 1) differentiating axial vs. non-axial CT images, 2) differentiating CXR vs. other radiographic imaging modalities, and 3) differentiating adult CXR vs. pediatric CXR. For the identification of individual OOD images, our framework achieved high sensitivity in detecting OOD inputs: 0.980 in CT, 0.984 in CXR, and 0.854 in pediatric CXR. Our framework is also adept at monitoring data streams and identifying the time a drift occurred. In our simulations tracking drift over time, it effectively detected a shift from CXR to non-CXR instantly, a transition from axial to non-axial CT within few days, and a drift from adult to pediatric CXRs within a day—all while maintaining a low false positive rate. Through additional experiments, we demonstrate the framework is modality-agnostic and independent from the underlying model structure, making it highly customizable for specific applications and broadly applicable across different imaging modalities and deployed ML models.

## Introduction

Machine learning (ML) is becoming prevalent in all aspects of healthcare due to its ability to recognize complex patterns in large datasets. However, this ability to learn from data can lead to instability and failure when ML models are exposed to data characteristics not represented in the training dataset.

When deployed in healthcare settings, the performance of ML models can change over time due to many reasons, including shifts in patient demographics or updates in data acquisition technology, but also due to the introduction of incorrect data types or labels into the input data stream [1]. Therefore, the importance of monitoring deployed AI models is becoming increasingly recognized as it offers a direct way to track AI systems post deployment [1]. Models can be monitored at either the input level, focusing on out-of-distribution (OOD) inputs, or at the output level, examining variations in the difference between the model output and the target variable. While several OOD detection methods have been discussed in the literature, most are tailored for the pre-deployment phase and often overlook drift monitoring aspects. Only a handful of studies [1–4] discussed using quality control management and temporal techniques for OOD detection and data drift monitoring. However, these studies mainly provide a theoretical foundation and do not discuss practical implementations. This work presents a practical implementation of a framework for data drift monitoring using Statistical Process Control (SPC) methods.

SPC is a well-known statistical method to monitor and control the quality of a production process by understanding sources of variation [5]. SPC control charts are visual statistical tools that can be used to easily detect changes in the process over time [5]. In this paper, we propose a framework for OOD detection and data drift monitoring that combines ML methods and geometric distances with SPC methods. This proposed framework is model and data source independent, and thus, has the potential for widespread adoption.

## Related Work

This section provides an overview of existing methods for OOD detection and data drift monitoring. OOD detection involves identifying samples that deviate from the expected in-distribution features, while data drift monitoring focuses on tracking these deviations over time. To learn the feature space of a dataset, several feature representation learning methods can be used.

### Feature Representation Learning

To identify images as OOD, they need to be converted into quantifiable feature representations. Several methods can be employed to extract these representations, including unsupervised learning methods, supervised learning methods, and contrastive learning methods.

Unsupervised learning methods do not require labels, as they learn based on underlying data patterns [6]. Generative models, which can be trained in an unsupervised manner, have been used for OOD detection [7, 8]. One specific example of generative models is the autoencoder, a neural network designed to learn feature representations by compressing the input data into a lower-dimensional representation (encoder) and then reconstructing the data back to its original form (decoder). Studies [9, 10] have demonstrated that autoencoders can extract discriminative features. While these methods have been employed to extract feature representations from images, it has been reported that they face challenges in reliably detecting OOD instances [9, 11], which is consistent with our findings in Section 4.

In contrast, supervised learning methods require labeled data to map input images to specific labels, making them effective for tasks where labeled data is available [6]. Supervised learning methods, such as convolutional neural networks (CNNs), can be used to train feature extractors that distinguish in-distribution images from OOD images. When explicit in-distribution and OOD labels are not available, dataset-specific labels can be used to train a feature extractor to extract relevant features from the dataset. Although supervised learning approaches have been widely used for feature representation, they can suffer from asymptotic overconfidence [12, 13]. This issue arises when networks, particularly those using ReLU activation functions, produce overly confident predictions for data points that are significantly different from their training data. Studies [13] have highlighted this issue. In such cases, the networks fail to “know when they don’t know”, leading to potentially dangerous situations where the model is overly confident. Uncertainty quantification (UQ) can address this issue by providing a measure of confidence. By quantifying uncertainty, we can identify when the model is unsure about its predictions, particularly for OOD inputs. Recent studies [14, 15] highlighted the importance of UQ in improving the reliability of OOD detection. These techniques could help ensure that models not only make accurate predictions but also recognize and express uncertainty when encountering unfamiliar data.

Another approach for extracting feature representation is contrastive learning, where the feature extractor is trained to differentiate between similar and dissimilar pairs of data [6]. By learning to distinguish between in-distribution and contrasting examples, the model can better identify OOD samples. One study [16] explored the impact of feature embedding on OOD strategies and found that feature representations extracted using contrastive learning can significantly enhance the performance of OOD detection. This finding is consistent with our results presented in Section 4.

### OOD Methods

After extracting feature representations, different metrics can be used to measure the distance between in-distribution and out-of-distribution data. Examples of well-known metrics include Mahalanobis distance [17], Euclidean distance [18], Geodesic distance [19], and cosine similarity [20]. In [17], the authors used the minimum Mahalanobis distance to all class centroids for OOD detection. Similarly, González et al. [21] used the Mahalanobis distance to detect OOD silent failures for Covid-19 lung lesion segmentation. Kore et al. [22] used the maximum mean discrepancy (MMD) for detecting distributional differences between source and target data. Other works have implemented geodesic (Fisher-Rao) distance [19] and cosine similarity [20] between test sample features and train features to determine OOD samples. It is important to note that non-parametric metrics (e.g., cosine similarity) do not impose distributional assumptions on the feature space, potentially offering more flexibility as compared to parametric metrics (e.g., Mahalanobis distance).

**Fig. 1:**
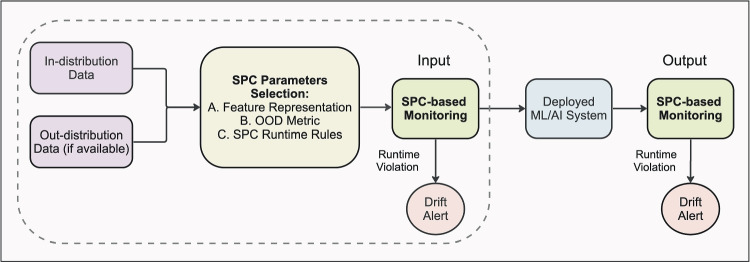
Overview of the application of the proposed SPC-based framework for drift monitoring. The dashed box indicates that this work focuses on monitoring inputs; however, the same framework can be applied to oversee the model’s outputs

In addition to distance-based metrics, other methods for OOD detection include outlier exposure strategies and one-class classification. Outlier exposure strategies involve exposing the training models to a set of collected OOD samples, or “outliers”, during training to help models learn in and out discrepancy [23]. One limitation of this method is the potential unavailability of OOD samples beforehand. In some practical scenarios, it might be challenging to obtain a representative set of outliers. One-class classification involves training a model to recognize a single class (in-distribution data) and then identify any data that does not belong to this class as OOD. It typically relies on training data from only one class without needing labeled OOD examples. For example, Ruff et al. [24] introduced a fully deep one-class classification method for anomaly detection. Their method trains a neural network to enclose the training data within a hypersphere in the output space, thereby learning the common factors of variation. Similarly, Wei et al. [25] used a one-class classification approach to detect OOD in medical images.

For a comprehensive review of other OOD methods, we refer reader to [26]. In addition, OOD detection challenges have been organized to compare and benchmark different OOD approaches. Among the notable efforts is the MICCAI “Medical Out-of-distribution detection challenge”, which provides a standardized dataset and benchmark for comparing different OOD detection approaches.

### Data Drift Monitoring

Although several methods have been proposed to identify data points that deviate from the distribution observed during training [27–37], current OOD detection methods in medical imaging are not designed to monitor such deviations over time. A research study [32] has shown that the performance of clinical prediction models trained on electronic health records (EHRs) degrades over time due to data drift resulting from distribution changes. Another research study [38], which analyzed the performance of ML models applied to 32 datasets from four different industries, found that 91% of these models suffer from performance degradation as they age due to several factors including change in data characteristic. This underscores the need to develop methods for monitoring drift over time to ensure the reliability of the data fed to ML models.

Several studies [3, 4, 39] have explored monitoring using Dynamic Adapting Window [39] and Adaptive Windowing (ADWIN) [40]. However, these methods have not been applied to monitor data drift in radiological images. Additionally, it is known that windowing-based methods tend to be less effective at detecting small, gradual shifts in a temporal signal. In contrast, cumulative sum (CUSUM), a type of SPC method, is well-suited for identifying small, gradual changes over time [41]. It offers greater stability and requires fewer computational resources.

While SPC was originally developed for industrial settings, SPC concepts have been introduced to healthcare settings for process improvement and quality control, such as in surgical care [42–44] and neonatal intensive care units [45]. Recently, Feng et al. [2, 46] discussed the need to adapt quality control methods for the monitoring of deployed AI systems. These prior works motivated us to adapt SPC methods for monitoring OOD in medical imaging applications. To our knowledge, this is the first work that provides a practical implementation of an SPC-based method for both OOD detection and input data drift monitoring in radiological images.

**Table 1:** Examples of parameters selection for designing SPC-based monitoring framework

Feature selection	Post hoc OOD metrics	SPC Tools/Rules
Unsupervised methods	Cosine similarity	$1,2,3\sigma$ SPC
Supervised methods	Mahalanobis distance	CUSUM
Contrastive learning methods		

The chosen features represent the incoming data stream; the post hoc OOD metrics are geometric distances to quantify changes in the feature space; and SPC tools formalized how OOD detection flags were thrown based on the calculated metrics

## Methods

This paper presents a ML-enabled SPC framework for OOD detection and data drift monitoring in radiological images. Figure 1 illustrates how the proposed framework can be integrated within ML development and used for data drift monitoring post deployment. In this paper, we focus on input monitoring, but this framework can be adapted for both input and output monitoring [41]. As shown in the dashed box in the figure, this framework begins with the selection of a dataset that accurately reflects the expected operating conditions (in-distribution) of the task followed by training a feature extraction model ($1^{st}$ column in Table 1). Optionally, a representation of the out-distribution data can also be provided if such data is known and available. Next, an appropriate post hoc OOD metric is chosen that quantifies the feature space and separates OOD inputs ($2^{nd}$ column in Table 1). Finally, SPC charts are generated using the computed OOD metrics ($3^{rd}$ column in Table 1). Each of these steps is described further below.

### Feature Representation

We investigated both unsupervised and supervised deep learning methods for training a feature extraction model. Monitoring features should be sufficiently sensitive and specific to relevant changes in the process being controlled. Due to the complex and hierarchical nature of the information in medical images, simple basic image statistics (e.g., mean, standard deviation, skewness, kurtosis) and texture features are insufficient to distinguish chest x-rays from other medical images types (refer to Figs. 6 and 7) using SPC methods. This baseline underscored the need to use more complex features derived from deep learning methods.

To extract relevant feature representations using supervised learning, we trained a convolutional neural network (CNN) with two methods: binary cross-entropy (BCE) and contrastive learning. We chose contrastive learning as a training method for its proven effectiveness in learning discriminative features [47]. This method focuses on identifying and capturing essential characteristics and similarities within the data, making it highly effective for detecting patterns and differentiating between various classes. After training task-specific models using binary cross-entropy and contrastive learning, we extract deep features from either the logits layer (in the CT task) or the final convolutional layer before the fully connected layer (CXR task). This supervised learning approach for extracting feature representation requires task-specific labels for training (e.g., healthy and abnormal labels in CXR).

For the unsupervised feature selection, we used an autoencoder to extract feature representation from unlabeled data. Details on the configurations, training procedures, and implementations for both supervised and unsupervised models are documented in Appendix A, Table 5 for autoencoder model, Table 6 for binary cross-entropy model, and Table 7 for contrastive learning model.

### OOD Metrics

The features extracted above are used to calculate distance-based metrics. To measure the distance between out-of-distribution samples and in-distribution samples, we used two geometric metrics: 1) Mahalanobis distance (MD) and 2) cosine similarity (CS). These metrics can be applied post hoc to a wide range of models due to their operation within the representation space, which enables them to bypass the need for model-specific adjustments (i.e., model-agnostic).

Mahalanobis distance (MD) is a parametric measure of the distance of a point from a distribution by considering the mean and the covariance matrix of the distribution. It estimates the probability density based on measurements from Gaussian distributed data, which is a reasonable data distribution assumption [48] for large and multi-dimension feature spaces. The formula [48] for the Mahalanobis distance $D_M$ of a point $\textbf{x}$ from a group of points with mean $\varvec{\mu }$ and covariance matrix $\textbf{S}$ is given by:

$$ D_M(\textbf{x}) = \sqrt{(\textbf{x} - \varvec{\mu })^\top \textbf{S}^{-1} (\textbf{x} - \varvec{\mu })} $$

Where $\textbf{x}$ is the vector of the point whose distance is being measured, $\varvec{\mu }$ is the mean vector of the points in the distribution, $\textbf{S}$ is the covariance matrix of the distribution, $\textbf{S}^{-1}$ is the inverse of the covariance matrix, and $(\textbf{x} - \varvec{\mu })^\top$ is the transpose of the difference between the point vector and the mean vector. The Mahalanobis distance accounts for the variance of each component (or feature) of the points and the covariance between components, making it a more robust distance metric in multivariate spaces compared to the Euclidean distance.

Cosine similarity (CS) is a non-parametric measure of similarity that calculates the cosine angle between two vectors $\textbf{A}$ and $\textbf{B}$ as [48]:

$$ \text {Cosine Sim}(\textbf{A}, \textbf{B}) = \frac{\textbf{A} \cdot \textbf{B}}{\Vert \textbf{A}\Vert \Vert \textbf{B}\Vert } $$

where $\cdot$ represents the dot product operator and $\Vert \cdot \Vert$ the vector magnitude. Cosine similarity ranges from -1 to 1. A value of 1 implies that the vectors are identical in orientation, 0 indicates orthogonality (no similarity), and -1 signifies that the vectors are diametrically opposed.

### SPC Implementation

SPC is a statistical method to monitor and control the quality of a process. It improves process quality through a better understanding of variation, differentiating between common sources of expected variation inherent to a process (common cause variation) and special sources of variation that can be eliminated [5].

We used SPC for OOD detection and data drift monitoring as follows. For each method of feature extraction, we calculated the mean $\mu$ and standard deviation $\sigma$ of the metric used for OOD detection (CS or MD) for the in-distribution training data. These metric statistics, $\mu$ and $\sigma$, establish the control limits for SPC analysis. We then computed a mean vector representing the average values of each feature across all in-distribution images. This mean vector is then used as a reference point to evaluate the distance metrics (OOD metrics), by comparing the feature vector of each test image against it. Moreover, for the Mahalanobis distance calculation, we derived the covariance matrix from the in-distribution data. Ultimately, a test image is identified as either in-distribution or out-of-distribution based on whether its computed OOD metric (CS or MD) falls outside the control limits defined by the $\mu$ and $\sigma$ of the training dataset.

We employed the three sigma ($3\sigma$) control charts for detection of individual OOD images. The $3\sigma$ control chart is a simple tool in which a process (i.e., an image) is considered out-of-control if the process outputs deviate beyond the range of $[\mu - 3\sigma , \mu + 3\sigma ]$, where $\mu$ denotes the mean and $\sigma$ denotes the standard deviation. In our study, we flag an individual test image or a day’s batch of test images (i.e., *N* images per day) as OOD if their OOD metric exceeds this $3\sigma$ range, determined by the mean and standard deviation of in-distribution images. Although we did not explore additional control limits in this work, it is important to note that additional run-time rules incorporating 1$\sigma$ or 2$\sigma$ can also be employed to accommodate the specific needs and sensitivities of different applications.

In addition to $3\sigma$ SPC, we employed CUSUM for monitoring data drift in input stream. CUSUM is a type of SPC chart utilized to monitor small shifts or changes in a process over time [49]. CUSUM plots the cumulative sum of deviations of each sample value from a target value, effectively highlighting trends or shifts. CUSUM calculates the cumulative sum of differences between individual data points and a target value or mean, expressed in its simplest form as a pair of equations [50]. The positive cumulative sum

1 $$\begin{aligned} S_i^+ = \max (0, S_{i-1}^+ + x_i - \mu _0 - k) \end{aligned}$$

and the negative cumulative sum

2 $$\begin{aligned} S_i^- = \min (0, S_{i-1}^- + x_i - \mu _0 + k) \end{aligned}$$

are the cumulative sums for detecting upward and downward shifts, respectively. Here, $x_i$ is the $i$-th individual data point, $\mu _0$ is the mean of the in-distribution process, and $k$ is an allowance, often set as $\frac{\sigma }{2}$. An alarm is triggered when either $S_i^+ > h$ or $S_i^- > h$, where $h$ is the decision interval or threshold, commonly set to $4 \times \sigma$. This decision rule implies that if either $S_i^+$ or $S_i^-$ exceeds $h$, a potential shift in the process has occurred, necessitating further investigation or action. The sensitivity of CUSUM is adjustable by modifying the allowance $k$ and the threshold $h$. Balancing CUSUM’s sensitivity involves a trade-off between false detection (type I errors) and detection delays or failures (type II errors). Therefore, setting CUSUM parameters involves striking a balance between minimizing false detection and accepting a tolerable delay in detection.

To assess the ability of CUSUM to monitor data drift, we created a simulation scenario spanning a time series from day 1 to day *N*, where each day comprises a batch of *d* images. We then computed a daily metric by averaging the OOD metrics (e.g., cosine similarity) across all images within each batch, thus representing the data of each day as a singular metric. Midway through the simulation-at day *N*/2-we introduced data drift by increasing the daily percentage of OOD inputs. This simulation is designed to evaluate the adaptability of our method to data pattern changes, mirroring real-world conditions where AI models might face variations from their initial training data once a certain period of time has passed.

**Table 2:** Comparison of OOD detection performance between the proposed SPC-based OOD detection, and one-class classification Method [24]

Task	One-class classification [24]		SPC-Based (Our method)	
	Sensitivity	Specificity	Sensitivity	Specificity
CT Task (axial CR vs. non-axial CT)	0.872	**0.924**	**0.985**	0.884
CXR Task (adult CXR vs. non-CXR)	0.907	0.962	**0.984**	**0.991**
CXR Task (adult CXR vs. pediatric CXR)	0.685	0.834	**0.854**	**0.851**

The best OOD performance is underlined and highlighted in bold

## Experiments and Results

We used publicly available CT exams and chest-x-ray (CXR) datasets to demonstrate the ability of our framework to identify OOD images and monitor drift in incoming data. Specifically, using CT exams, we demonstrate the ability to differentiate between axial vs. non-axial CT images. Using CXR datasets, we successfully demonstrated our method’s capability to identify different types of drift in the data. This includes distinguishing changes in imaging modalities (from CXR to other radiographic images), demographic shifts (from adult to pediatric CXR images), and variations in the data source (from NIH CXR dataset to PadChest CXR dataset).

We structured the results of CT and CXR tasks into a four-pronged description of: 1) the used datasets, 2) the process of selecting SPC parameters, 3) OOD image detection results, and 4) OOD monitoring results. In the monitoring experiments for all tasks, we assumed that the duration of monitoring spanned two months ($N=60$) and that each day involved the analysis of 100 images ($d=100$) per day.

For all tasks, we compared our method to the state-of-the-art Deep One-Class Classification for OOD detection [24]. This method trains a neural network to minimize the volume of a hypersphere that encloses the network representations of the data. It extracts common factors of variation by mapping data points close to the center of the sphere. For our comparison, the normal class represents in-distribution data, and the abnormal class or anomaly is considered OOD. We used the implementation provided by the authors on Deep-SVDD-PyTorch to generate the results for CT and CXR tasks. The results in Table 2 demonstrate that our SPC method achieved higher sensitivity (i.e., a higher rate of detecting OOD) compared to the one-class classification method for CT and CXR tasks.

### CT Task: OOD Detection and Drift Monitoring

The OOD CT task is designed to distinguish between axial CT images (in-distribution) and coronal/sagittal CT images (out-of-distribution). This task is motivated by the fact that ML models in CT analysis are typically trained on specific image orientations; therefore, multiplanar reformatted images, which deviate from the training distribution, might lead to reduced performance. In this scenario, an OOD detection model is trained on all axial CT images, thus axial CT images define in-distribution data. After training, the model was trained on a stream of data consisting of a mix of new unseen in-distribution data - axial CT images - and new OOD CT images of different views - coronal and sagittal.

#### Dataset

**Fig. 2:**
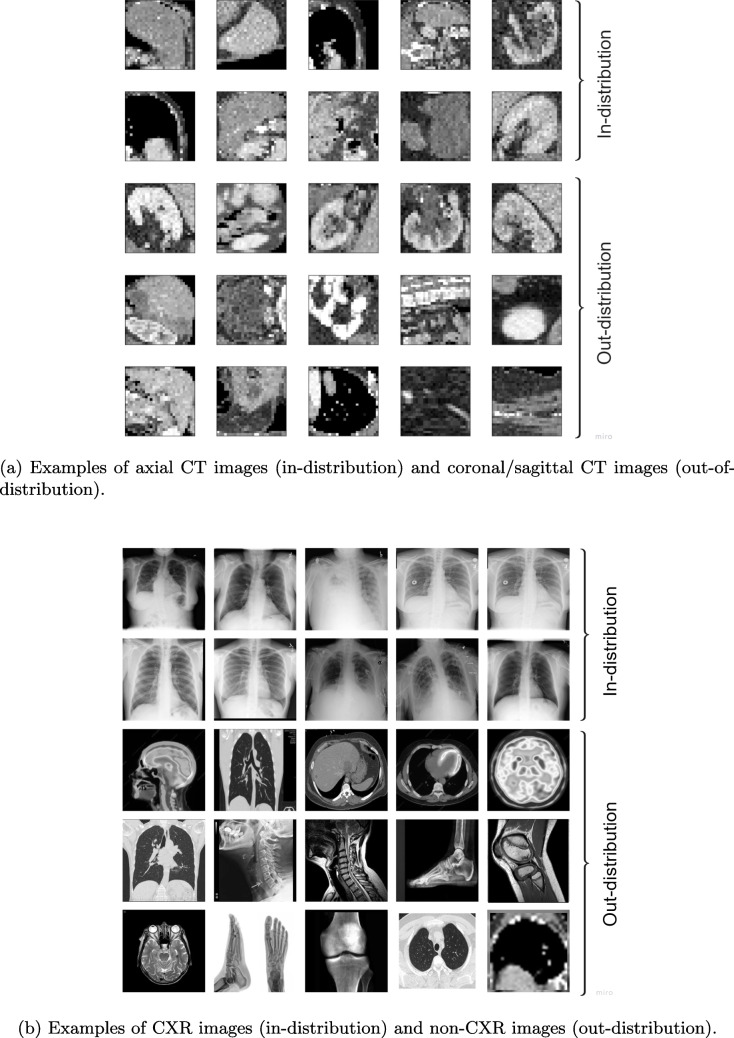
Examples of in-distribution and out-of-distribution images from the datasets used in this study

We used CT images from the open-source Medical MNIST dataset [51], which contains 58,850 samples. Specifically, we used OrganMNIST subset of the Medical MNIST dataset; the dataset authors sliced 3D CT scans (from the Liver Tumor Segmentation Benchmark (LiTS) [52] along three different viewing planes – axial, coronal, and sagittal – and pre-processed them into $28\times 28$ grayscale images, similar to digit MNIST. All the images were normalized ($\mu =0.5$, $\sigma =0.5$) prior to model training. Figure 2a shows examples of in-distribution (axial) and out-of-distribution (coronal/sagittal) images for this dataset. We note that we used CT slices as the unit of analysis in our study as patient-level data are not available.

#### Model Specifications and Training Details

We compared three feature extraction models: 1) an unsupervised convolutional autoencoder, 2) a slice-supervised binary cross-entropy (BCE) ResNet-18, and 3) slice-supervised contrastive ResNet-18. All these models were trained using the training and validation splits created by the MedMNIST authors [51]. All models were trained using in-distribution and out-of-distribution labels, based on the assumption that OOD labels are available in some cases; i.e., we have knowledge of what constitutes OOD data. We refer the reader to Tables 5, 6, and 7 in Appendix A for more details about the hyper-parameters for all three feature extraction models.

**Table 3:** Performance of $3\sigma$ OOD detection of non-axial CT slices for each feature representation and metric

OOD metric	Unsupervised autoencoder		Supervised BCE ResNet-18		Contrastive ResNet-18 [16]	
	Sensitivity	Specificity	Sensitivity	Specificity	Sensitivity	Specificity
Cosine	0.014	0.989	0.980	0.830	**0.985**	**0.884**
	[0.002-0.029]	[0.976-1.000]	[0.969-0.996]	[0.789-0.870]	[0.960-0.996]	[0.801-0.884]
Mahalanobis	0.003	0.999	0.977	0.849	0.966	0.849
	[0.000-0.012]	[0.996-1.000]	[0.959-0.992]	[0.805-0.889]	[0.942-0.989]	[0.805-0.895]

$95\%$ confidence intervals were calculated by bootstrapping with $n=100$ samples over test-set subsets of size $m=500$. The best performance is underlined and highlighted in bold

#### Results for SPC-based OOD Detection

Table 3 reports the image-level OOD detection performance across different feature extraction methods and distance metrics. We calculated the performance (sensitivity and specificity) with bootstrapped confidence intervals ($n=100$) over random sub-samples ($\text {size} = 500$) of the test set. Images were flagged if their OOD metrics fell outside the $\mu \pm 3\sigma$ control limits. While the autoencoder resulted in the smallest mean sensitivity (CS: 0.014; MD: 0.003), it yielded the highest mean specificity (CS: 0.989; MD: 0.999). The low sensitivity and high specificity for the autoencoder could be explained by its feature representation: incoming images all had features with similar distances from the training distribution, making OODs hard to distinguishfor $3\sigma$ flagging. Thus, the model flagged only a few samples as OOD, yielding the reported low sensitivity and high specificity.

**Fig. 3:**
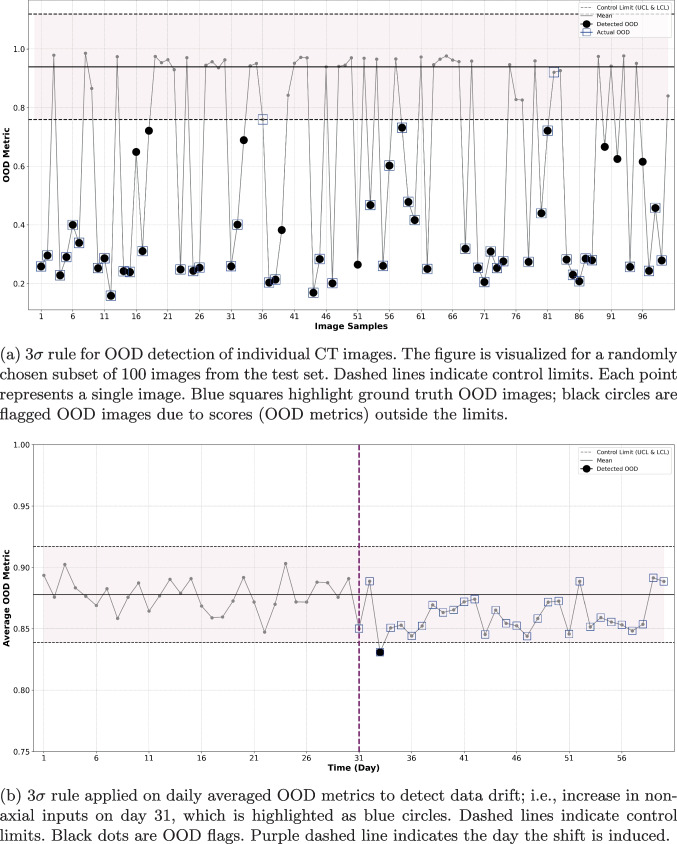

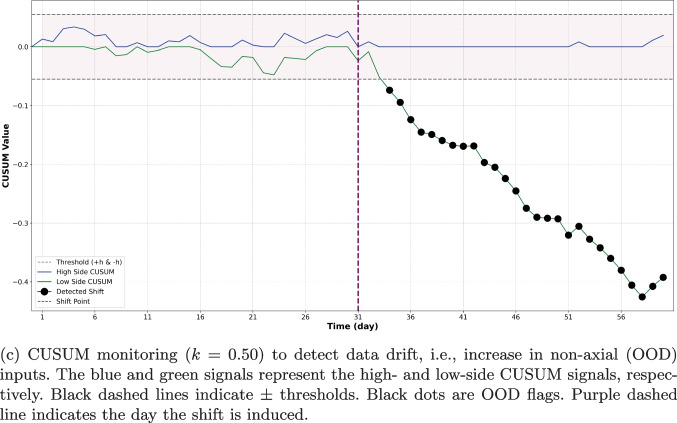
SPC-based detection of OOD non-axial CT slices and monitoring for data-drift. All experiments tracked the cosine similarity of the test features to the training in distribution features. SPC-based detection of OOD non-CXR images and monitoring for data-drift. All experiments tracked the cosine similarity of the test features to the training in-distribution features

Supervised BCE ResNet-18 significantly improved accuracy and sensitivity compared to the unsupervised baseline ($p < 0.01$ for all comparisons). Among the supervised methods, contrastive ResNet-18 features with cosine similarity resulted in the highest mean accuracy (CS: 0.913) and specificity (CS: 0.848). Mean sensitivity was similar between contrastive (CS: 0.980) and BCE ResNet-18 (CS: 0.985) features. Figure 3a visualizes the image-level $3\sigma$ cosine similarity detection of contrastive ResNet-18 features. The figure illustrates that most OOD images are identified with a high degree of confidence, evidenced by their large difference compared to the mean cosine similarity. Therefore, given the favorable sensitivity-specificity tradeoff and robust in- vs. out-distribution separation in feature space, we used contrastive ResNet-18 features and the cosine similarity metric for the subsequent OOD monitoring experiment.

#### Results for SPC-based OOD Monitoring

In our CT monitoring simulation, we assumed a clinical facility collects 100 abdominal CT slices per day. During the first month, we set the daily OOD percentage uniformly between 0-1%. In the second month, we increased the OOD rate to be uniformly sampled from 3-5%. Figure 3b and c highlight the difference in OOD sensitivity between $3\sigma$ and CUSUM. The $3\sigma$ monitoring throws a flag on day 34; most subsequent days are close to the lower $3\sigma$ control limit, but are within the control limits. On the other hand, CUSUM monitoring reports a flag two days after the induced shift. The low-side CUSUM signal significantly decreases after this day, lending support to the initial flag. The parameter *k* can also be tuned to reduce false positive rate or detection delay (see Table 8).

**Table 4:** Performance of $3\sigma$ OOD detection of non-CXR images for each feature representation and distance metric

OOD Metric	Unsupervised Autoencoder		Supervised BCE VGG16		Contrastive VGG16 [16]	
	Sensitivity	Specificity	Sensitivity	Specificity	Sensitivity	Specificity
Cosine	0.912	0.944	**0.984**	**0.991**	0.932	0.948
	[0.896-0.943]	[0.922-0.964]	[0.974-0.989]	[0.981-0.987]	[0.933-0.948]	[0.954-0.965]
Mahalanobis	0.905	0.951	0.971	0.985	0.929	0.943
	[0.868-0.916]	[0.907-0.957]	[0.959-0.980]	[0.967-0.988]	[0.928-0.933]	[0.929-0.957]

$95\%$ confidence intervals were calculated by bootstrapping with $n=100$ samples over test-set subsets of size $m=500$. The best OOD performance is underlined and highlighted in bold

### CXR Tasks: OOD Detection and Drift Monitoring

With CXR images, we focused on distinguishing between CXR images and non-CXR images as well as adult CXR images from pediatric CXR images. In all experiments, we operate under the assumption that developers do not have knowledge (*a priori*) about the potential characteristics of OOD images; their familiarity is limited to the CXR images and labels utilized during the model’s training phase; and therefore, we used disease-specific labels for building the feature extraction model.

#### Dataset

We used the NIH CXR dataset [53] as our training dataset to define in-distribution images. The NIH CXR dataset contains 112,120 frontal-view X-ray images with no-findings and fourteen disease labels extracted from corresponding radiological reports. For this task, we assumed that out-distribution data is unknown, and thus we approached feature extraction by training a model trained to classify CXR images as either healthy (no findings) or abnormal (disease labels). However, it is important to note that although we used a binary classification model, other models (e.g., multi-label or pneumonia detection), could also be utilized effectively for learning and extracting in-distribution feature representations without knowing the nature of OOD data.

We used three additional datasets to represent OOD inputs. The first testing set contains non-CXR radiological images such as CT images and bone X-ray images (see samples in Fig. 2b). The inclusion of other imaging modalities enabled us to test the ability of the method to detect out-of-modality data. The second test set contained pediatric CXRs from the open-source Pediatric Pneumonia Chest X-ray dataset [54], which contains 5,856 Chest X-rays labelled as either pneumonia or normal. For examples of pediatric CXR images, refer to Fig. 8 in Appendix A. This pediatric dataset enables the assessment of how well our approach, trained on adult CXR, can identify demographic changes and flag the pediatric set as OOD. The third dataset is another CXR test called PadChest dataset [55], which contains over 160,000 images from 67,000 patients interpreted by radiologists at Hospital San Juan (Spain). This PadChest dataset enables the assessment of our approach in detecting shifts related to images coming from another dataset source/origin. These test sets enable us to investigate three clinically relevant scenarios: out-of-modality, within-modality, and cross-dataset.

All images from the training and testing sets were resized to $256 \times 256$ to meet the requirements of the model (VGG16).

#### Model Specifications and Training Details

We compared three feature extraction methods (Section 3.1): 1) an unsupervised convolutional autoencoder, 2) a supervised binary cross-entropy (BCE) VGG16, and 3) supervised contrastive VGG16. Models were trained using disease labels (i.e., healthy vs. abnormal) readily available in the dataset. Without explicit OOD labels, we hypothesize that utilizing disease-specific labels to learn in-distribution image features can facilitate distinguishing between the feature spaces of in-distribution and OOD (non-CXR) images. We trained and validated the feature extraction model using 80% and 20% of NIH dataset, respectively. Details about the models’ hyper-parameters can be found in Tables 5, 6, and 7.

#### Results for SPC-based OOD Detection

**Fig. 4:**
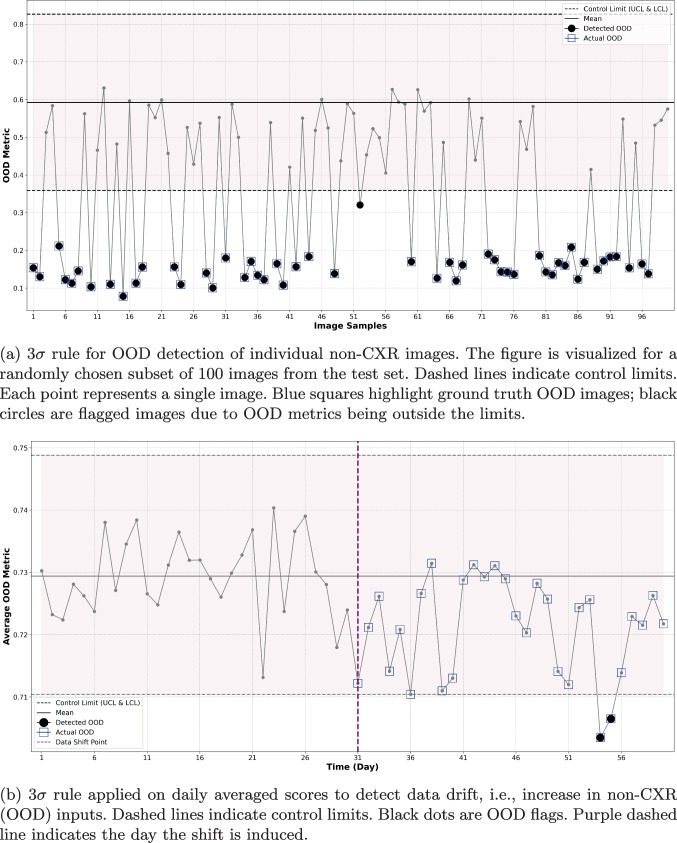

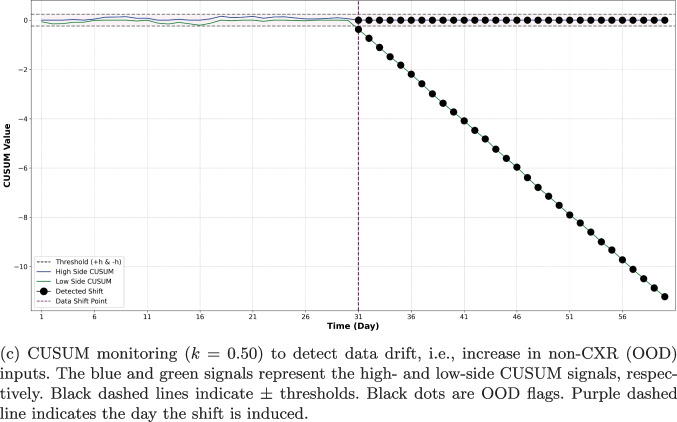
SPC-based detection of OOD non-CXR images and monitoring for data-drift. All experiments tracked the cosine similarity of the test features to the training in-distribution features

Table 4 reports OOD detection performance. Although we observed variations in performance across the feature extraction methods and distance metrics, all methods achieved acceptable performance. Notably, the supervised VGG16 demonstrated superior sensitivity (CS: 0.984) compared to the unsupervised baseline, with all values reported within a 100-sample bootstrapped 95% confidence interval. Interestingly, unlike in CT task (Table 3), the supervised BCE-loss VGG16 outperformed the model trained with contrastive-loss. In all results within Table 4, feature quantification using cosine similarity generally yielded better performance than when using the Mahalanobis distance. We can also observe that unsupervised learning method for feature extraction (i.e., autoencoder) achieved the lowest sensitivity. Figure 4a shows how the proposed SPC-based method successfully flagged randomly selected 100 images as OOD; note that each point here represents the OOD metric computed for a single image.

To further probe the performance of the proposed method, we conducted two additional experiments focusing on cross-modality and cross-dataset evaluation.

For the first additional experiment, we conducted subgroup analysis on modality type to report the performance in detecting various modalities individually. Our findings indicate that our method exhibited robust OOD detection across all modalities in the test set, achieving an accuracy $>0.98$ and a sensitivity $>0.96$. These results suggest that the proposed method is modality independent. Specifically, our method showcased the ability to flag various imaging modalities (i.e., CT, MRI, bone X-ray, and ultrasound) as OOD compared to the CXR modality. This ability to recognize images as OOD, irrespective of the modality, offers an advantage as it eliminates the need for training separate models for each modality to classify them as OOD.

In addition, we conducted an experiment to assess the performance of our proposed method across different datasets, specifically to evaluate its ability to detect subtle drifts within the same modality. To achieve this, we established control limits of SPC using the NIH CXR dataset followed by testing using images from the PadChest CXR dataset [55]. This enables determining if the method could detect subtle changes in dataset characteristics, considering the common issue of performance degradation when transferring models from one CXR dataset to another due to shortcut learning, as observed in [56]. Our method demonstrated a sensitivity of 0.852 and a specificity of 0.826. These results suggest that the SPC-based method can effectively identify subtle drifts across datasets.

#### Results for SPC-based OOD Monitoring

In our CXR monitoring simulation, we assumed a clinical facility receives 100 images per day, and during the first month, we set the daily OOD rate to uniformly vary between 0-1%. In the second month, we increased the OOD rate to be uniformly sampled from 2-4%.

Figure 4b and c illustrate the performance of both $3\sigma$ and CUSUM monitoring methods. A comparison of Fig. 4a with Fig. 4b reveals that the $3\sigma$ SPC chart effectively flagged individual OOD images, yet its performance in identifying OOD batches was less efficient, as shown in Fig. 4b. However, it is observable that several points post day 30 consistently fell below the mean and approached the lower $3\sigma$ control limit. This pattern suggests that these points would be flagged as OOD under different runtime rules, such as if two out of three consecutive points lie beyond the $2\sigma$ limit. In contrast to the daily average $3\sigma$, the CUSUM method, depicted in Fig. 4c, successfully identified the shift that occurred on day 31, without a delay when a k-value of 0.5 was used. See Table 8 in Appendix A for an evaluation of the performance of the CUSUM method across a range of k values.

##### Monitoring a Demographic Drift in CXR Images

**Fig. 5:**
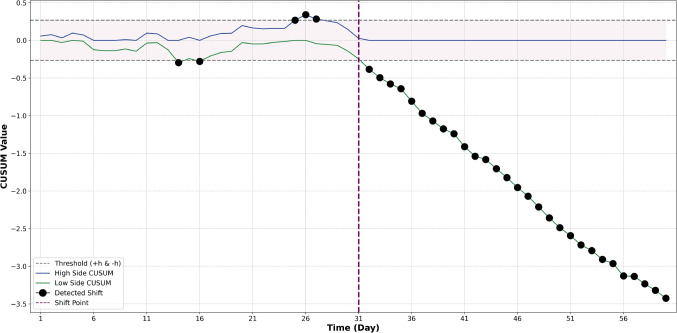
CUSUM monitoring ($k=0.50$) for distributional shift towards pediatric CXR inputs. The blue and green signals represent the high- and low-side CUSUM signals, respectively. Black dashed lines indicate ± thresholds. Black dots are OOD flags. Purple dashed line indicates induced shift on day 31

**Fig. 6:**
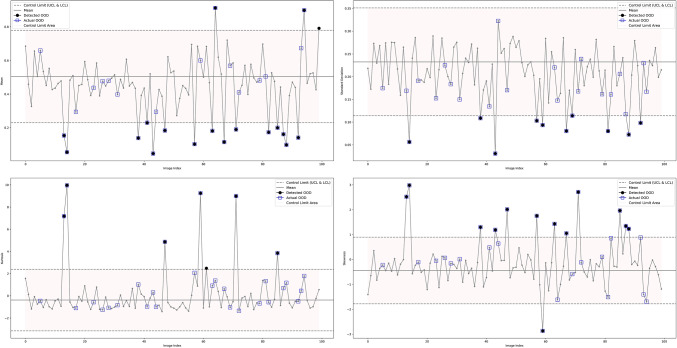
$3\sigma$ OOD detection of non-CXRs using simple image statistics. Four zero-order features (mean, standard deviation, kurtosis, and skewness) were evaluated for OOD detection. The black dots represent the points flagged as OOD as they are outside the control limits. The blue squares represent the images that are known to be non-CXR (ground truth OOD). From all the figures above, we can observe low accuracy in flagging OOD points with 0.57, 0.33, 0.43, 0.20 for mean, standard deviation, skewness, and kurtosis, respectively. The performance of OOD detection after combining all four statistics was $<0.50$, which is much lower than using deep learning features. This highlights the impact of the feature representation on the performance

**Fig. 7:**
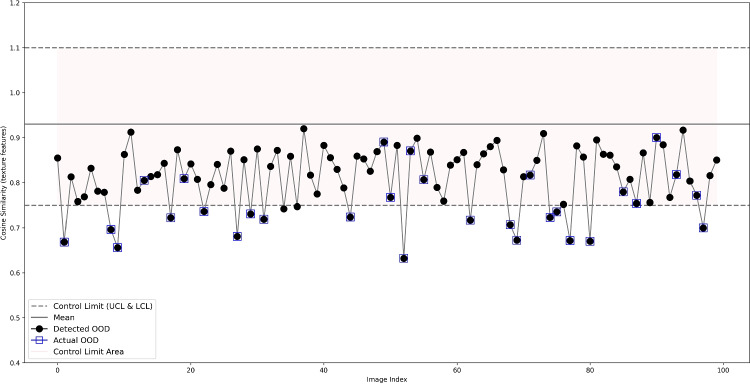
$3\sigma$ OOD detection of non-CXRs using texture features. We utilized grey level co-occurrence matrices (GLCM) to quantify texture information, recording the frequencies of adjacent pixel pairs in an 8-bit image. By averaging these matrices across the eight nearest neighboring pixels, we accounted for rotational variations. From these averaged GLCMs, we derived the following features: contrast, correlation, energy, and homogeneity. Each of these features offers insights into different aspects of texture. These texture features combined represent the feature representation. The black dots that exceed the control limits indicate the points flagged as out-of-distribution (OOD), as they lie outside the specified control limits. The blue circles represent the images that are known to be non-CXR (ground truth OOD). In this figure, we observe that 12 out of the 30 actual OOD images were not correctly identified

**Fig. 8:**
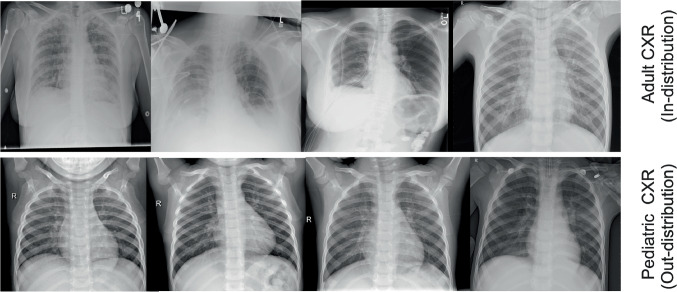
Examples of Adult CXR images (in-distribution) and pediatric CXR images (out-distribution)

We also assessed whether our method could effectively monitor and flag changes in the demographic characteristics of patients’ imaging data, such as age group variations. Our results showed that the proposed method can detect pediatric CXRs as OOD. Our method successfully detected this distribution change with an accuracy of 0.852, sensitivity of 0.854, and specificity of 0.851. We also found that CUSUM rapidly detected the demographic change as shown in Fig. 5. However, we can observe false positive flags prior to the induced shift. This might arise partly because the used pediatric dataset includes a wide age range, from infants to 18-year-olds, making some pediatric CXR images very similar to those in the adult training set (see Fig. 8 in Appendix A). Nonetheless, the proposed method maintains acceptable performance in identifying the induced shift-specifically, the incorporation of 3-5% pediatric CXR into the daily batch (*N*=100 images) of CXR images.

## Discussion

This is the first work we are aware of that combines ML methods and geometric distances with SPC methods to provide a practical solution for OOD detection and data drift monitoring in radiological imaging. We provide open access to our implementation of the framework and the datasets online at [GitHub].

Through a series of experimental scenarios, we demonstrate the effectiveness of the proposed framework across various imaging modalities and use cases, demonstrating that our framework is modality-agnostic and capable of detecting data drift, irrespective of the dataset source.

For the CT imaging modality, we demonstrated the ability to differentiate between axial and non-axial CT images in an input stream of CT images. Labelled in- and out-of-distribution images were available to train the monitoring algorithm for this task. In the second scenario, labeled in- and out-of-distribution examples were not available to train a CXR monitoring algorithm to detect non-CXRs or demographically shifted CXRs in a primarily adult CXR input stream. A central difference between these tasks is the access to supervised labels during training. The feature extraction for the axial CT monitoring task necessitated training on a repository of slice-supervised CT images; therefore, as we show in Table 3, the best features for the CT task were derived from slice-supervised contrastive training. In contrast, we did not know *a priori* what the OOD distribution would look like in the CXR task. The OOD images in the test set consisted of medical images of diverse modalities, including ultrasound, MRI scans, and bone X-rays, as well as pediatric CXR. Thus, the optimal feature space representation relied on training with the disease pathology labels and using a vector similarity metric to flag OOD data (see Table 4). Through these experiments, we successfully demonstrated that our proposed framework can be applied in both scenarios. Additionally, our framework can be easily extended to other medical imaging applications as well as other modalities and can possibly also be used as an approach for open set recognition, where unknown classes are flagged as OOD [57, 58].

With both medical imaging modalities, we illustrate the strengths of using different SPC schemes for different tasks: $3\sigma$ is effective at image-level OOD detection, but CUSUM is better suited for monitoring data drift. With $3\sigma$, we set the mean $\mu$ and standard deviation $\sigma$ of the distance metric in feature space based on the training set. At runtime, individual images were flagged if their OOD metric exceeded $3\sigma$. This method showed high specificity and sensitivity in individual OOD image detection (Tables 3 and 4, Figs. 3a, and 4a). However, it struggled to flag temporal drift in batched input streams (Figs. 3b and 4b), as daily averaging of scores tended to obscure anomalous deviations in the distance metrics. In contrast, CUSUM, which accumulates data drift, was more effective in these scenarios (Figs. 3c and 4c), consistently detecting induced shifts after only a few days. Our findings highlight the importance of selecting control metrics specific to the task at hand. Similarly, we demonstrate that the choice of features and OOD metrics are task-specific and the efficacy of the SPC-based method in detecting OOD changes is contingent on the chosen features and their quantification. This is unsurprising, as different clinical scenarios have different sensitivity and specificity operating points as well as different data availability for model training, which is required for defining the in-distribution that sets the control limit.

The ability of CUSUM to detect the addition of pediatric data to a stream of adult CXR data illustrates the challenge of interpreting flagged data drifts. While it is tempting to conclude that the drift is related to patient demographics, such a conclusion is not warranted. Our proposed method has shown great utility in flagging data drifts; however, our method does not identify the source of those data drifts. For example, the drift observed in (Fig. 5) may be caused by a variety of factors–including patient demographics, site location, image acquisition techniques, or others. Our proposed method is efficient at identifying that something has changed, hence allowing further inquiry into what exactly has caused the drift, but does not reveal the underlying causes for the change.

SPC-based OOD detection offers several advantages over previous OOD detection methods (see Section 2). Unlike OOD detection that relies on fixed thresholds based on prior assumptions or empirical data, our framework uses SPC charts to dynamically adjust thresholds based on real-time data statistics. This adaptation ensures that our SPC-based OOD method stays effective even as data distributions evolve over time. By continuously monitoring and plotting metrics, our method can detect subtle shifts and data drifts that fixed thresholds might miss. This ongoing assessment allows for immediate adjustments, keeping the system relevant and effective in real-world applications. In addition, as SPC methods are rooted in statistical concepts, they are generally applicable to all monitoring scenarios where data is approximately Gaussian distributed, which is a reasonable assumption for most real-world applications of sufficiently large sample sizes. This enables the application of SPC-based OOD to a broad spectrum of data sources including CT and CXR monitoring as demonstrated in this work. It makes our framework versatile and broadly applicable in diverse data drift monitoring contexts.

While our experiments demonstrated promising results in detecting OOD images and monitoring data drift, our work has limitations that we plan to address in future work. One limitation is the reliance on simulated temporal data, which may not fully capture the complex and unpredictable nature of data drifts encountered in real-world clinical environments. We stress that in the current work, our goal was providing a proof of concept; in future work, we plan to explore the use of our data drift monitoring framework across multiple clinical sites that have real-patient datasets with associated metadata and actual timestamps. Another limitation is that our study did not investigate corrective actions that could be implemented after detecting data drift. We omitted this analysis here because the purpose of this study was to show that an SPC-based monitoring scheme could indeed flag when data were OOD. In future work, we will explore the efficacy of strategies such as model retraining and recalibration.

Additionally, in the current study we considered slices as the unit of analysis, which introduces another limitation. While we believe this risk is minimal for our specific screening function, where the model was designed to screen out non-axial slices rather than diagnose pathology, the limitation is inherent in using slices rather than whole scans. In the case of the OrganMNIST CTs, patient-level data (and whole case data) were not accessible, which limits our ability to account for slice correlations. In our future work, we plan to base our experiments on real patient longitudinal clinical data, which would allow us to better address patient-level correlations and improve the robustness of our findings. Further, our study does not discern possible underlying causes for the flagged drifts. In real-world, many complex factors could contribute to changes in the input distribution (e.g., image acquisition, image preprocessing, and patient demographics). To address these issues, we plan to integrate methods that can pinpoint specific features that contribute to OOD. Additionally, incorporating metadata as a prior input will provide deeper insights into the context of data shifts, helping to identify potential external factors influencing changes. The exploration of both OOD causes and corrective actions can advance our management of data drift in real-world scenarios. Finally, we plan to extend our current feature representation approach to explore an ensemble approach and investigate advanced feature extraction methods that can account for the complex geometry of the latent space.

## Conclusions

We introduce a new framework for OOD detection and data drift monitoring. Specifically, we use machine learning feature extraction methods to track deviations from training data, employing geometric metrics such as cosine similarity and Mahalanobis distance to quantify differences. By using SPC charts to visualize these metrics, we demonstrate the ability to efficiently and promptly flag OOD inputs.

Our results demonstrate the effectiveness of our framework in identifying OOD images, such as incorrect CT views, non-CXR images, and OOD images related to changes in demographic or data characteristics of CXR across datasets. This capability is not just an academic exercise but a practical tool in addressing the real-world issue of data drift in clinical settings. The mislabeling errors of medical imaging exams, as evidenced by previous research [31], can have significant repercussions, including the misapplication of ML models to inappropriate studies. This research, therefore, serves as a bridge between theoretical ML advancements and the pressing needs of the healthcare industry, highlighting the critical role of quality assurance in the deployment of AI technologies.

## Appendix A

**Table 5:** Summary of autoencoder parameters for CT and CXR applications

Parameter	Value
Number of epochs	10 (CXR), 100 (CT)
Loss function	Mean squared error loss
Optimizer	AdamW
Learning rate	0.001
Hidden channels (c_hid)	16
Latent dimension	100
Encoder conv layers	5 Conv2d, 5 ReLU
Decoder conv layers	5 ConvTranspose2d, 5 ReLU

**Table 6:** Summary of parameters in supervised binary cross-entropy learning for CT (ResNet-18) and CXR (VGG16) applications

Parameter	Value
Optimizer	Adam (learning rate = 0.0001 (CXR), 0.001 (CT))
Loss function	Binary Cross-Entropy
Number of epochs	100 (CXR), 100 (CT)
Batch size	32 (CXR), 128 (CT)

**Table 7:** Summary of parameters in supervised contrastive learning for CT (ResNet-18) and CXR (VGG16)

Parameter	Value
Loss function	Supervised Contrastive (SupConLoss) [59]
Temperature for loss	0.07
Training approach	Batch-wise training with accumulated loss
Optimizer	Adam (learning rate = 0.0001 (CXR), 0.001 (CT))
Batch size	32 (CXR), 128 (CT)

**Table 8:** Impact of CUSUM parameter *k* on detection delay (measured in days after day 31) and false positives (FP)

*k*	Delay (CT)	FP (CT)	Delay (CXR)	FP (CXR)
0.10	2	0	1	2
0.25	3	2	2	1
0.50	4	0	2	0
0.75	4	3	2	0
